# Unravelling the Biology of EhActo as the First Cofilin From *Entamoeba histolytica*

**DOI:** 10.3389/fcell.2022.785680

**Published:** 2022-02-25

**Authors:** Nitesh Kumar, Pragyan Parimita Rath, Priyanka Aggarwal, Sankar Maiti, Neel Sarovar Bhavesh, Samudrala Gourinath

**Affiliations:** ^1^ Department of Pathology, Indira Gandhi Institute of Medical Sciences, Patna, India; ^2^ International Centre for Genetic Engineering and Biotechnology, New Delhi, India; ^3^ School of Life Sciences, Jawaharlal Nehru University, New Delhi, India; ^4^ Indian Institute of Science, Education and Research, Kolkata, India

**Keywords:** actophorin, *E. histolytica*, NMR structure, actin-depolymerising factor, phagocytosis, cofilin

## Abstract

Actin-depolymerising factors (ADF) are a known family of proteins that regulate actin dynamics. Actin regulation is critical for primitive eukaryotes since it drives their key cellular processes. *Entamoeba histolytica*, a protist human pathogen harbours eleven proteins within this family, however, with no actin depolymerising protein reported to date. We present here the NMR model of EhActo, the first Cofilin from *E. histolytica* that severs actin filaments and also participates in cellular events like phagocytosis and pseudopod formation. The model typically represents the ADF-homology domain compared to other cofilins. Uniquely, EhActo lacks the critical Serine3 residue present in all known actophorins mediating its phospho-regulation. The second mode of regulation that cofilin’s are subjected to is via their interaction with 14-3-3 proteins through the phosphorylated Serine residue and a consensus binding motif. We found a unique interaction between EhActo and 14-3-3 without the presence of the consensus motif or the phosphorylated Serine. These interesting results present unexplored newer mechanisms functional in this pathogen to regulate actophorin. Through our structural and biochemical studies we have deciphered the mechanism of action of EhActo, implicating its role in amoebic biology.

## Introduction

Cellular movement depends on the actin cytoskeletal dynamics regulated by various proteins. Actin is subjected to several regulatory controls for precisely sculpting its function. This job has been assigned to several families of proteins that modulate actin dynamics. One of the widely studied regulators is the ADF/Cofilin (AC) superfamily, with its numerous members across all clades of organisms. AC proteins drive the motility and mechano-sensing activities in the cell (Narita, 2020). They restructure actin filaments continuously by both increasing the monomer dissociation, as well as severing the filaments (Kanellos and Frame, 2016). The implications of these protein interactions are of greater importance in single-celled organisms, such as the pathogenic *Entamoeba histolytica*. Thus, understanding the biological ramifications of AC proteins would unravel the effects of actin depolymerisation working in them.

AC family is recruited in the cell to recycle actin monomers (Bamburg and Bernstein, 2008). Some members accelerate monomer dissociation from pointed F-actin ends. They also bind twisted actin filaments to stabilize the kink, thereby augmenting their severing activity (Bernstein and Bamburg, 2010). Newer filaments ends are generated in the process that can be used for speedy polymerisation (Van Troys et al., 2008). If the actin monomer is largely sequestered, then the severing activity leads to a rapid decline in F-actin structures (Xue and Robinson, 2013). Thus, in conjunction with other regulators, ACs can function in increasing and decreasing F-actin in the cell.

Actophorin was identified as the first amoebic cofilin in *Acanthamoeba castellani* (AcActo) (Cooper et al., 1986). It was named the “actin carrying” protein that bound both actin forms (Maciver et al., 1991b; Blanchoin and Pollard, 1998; 1999). It interacts strongly with ADP-actin while mediating a weaker interaction with ATP bound monomer (Maciver and Weeds, 1994). Further, actophorin and profilin share overlapping binding sites for actin, thus, participating in a competition for the same (Blanchoin and Pollard, 1998). Nonetheless, profilin and actophorin work synergistically to rapidly recycle actin monomers to add on to new branches created by the Arp2/3 complex (Blanchoin et al., 2000a). Actophorin was proposed to bind along the actin filaments with a predilection for bent F-actin nooks in a pH-insensitive manner (Maciver et al., 1991b). It accelerates the dissociation of γ-phosphate from F-actin to further promote de-branching (Blanchoin et al., 2000a). Surprisingly, amoebic Ca^2+^-F-actin was resistant to actophorin, while Mg^2+^-F-actin was rapidly severed by it (Mossakowska and Korn, 1996). Phosphorylation of the first Serine residue (designated as Ser1 in AcActo and Ser3 in vertebrates) by LIMK activated by PAK directly inhibits any actin interaction (Blanchoin et al., 2000b). In a moonlighting function, actophorin forms rigid α-actinin bound actin bundles that are less thixotropic (Maciver et al., 1991a).

Multiple regulatory factors control AC activity in the cells. Phosphorylation and de-phosphorylation of Ser3 (vertebrate nomenclature) are one of the primary regulation circuits. Phospho-AC (p-AC) is the deactivated form such that it can neither bind actin nor sever it (Agnew et al., 1995; Pederson, 2008). Ser3 is present at the binding interface of AC-actin; thus, the phosphorylation blocks binding either through steric hindrance or by electrostatic repulsion with the net negative surface of actin (Narita, 2020). Studies conducted on S3D mutant (mimics the p-AC form) revealed the mechanistic details of how the p-AC is rendered inefficient for severing activity (Elam et al., 2017). The second regulatory molecule showcased is 14-3-3, a ubiquitous protein family present in almost all cells. 14-3-3 regulates actin dynamics through various interactions with LIMK, TESK, SSH, and cofilin (Ohashi, 2015). 14-3-3 binds to the phosphorylated forms of these proteins and affects their functionality. A recent study reported, 14-3-3 recruited EhCoactosin during phagocytosis (Agarwal et al., 2019). However, the 14-3-3 binding sites in EhCoactosin were not the canonical ones.

The current study characterizes *Entamoeba histolytica* actophorin (EhActo) through various approaches. We have cloned and purified EhActo to characterise its functional attributes. Our data reveals EhActo as the first true cofilin from *Entamoeba*, unlike the role reversal activity presented by EhCoactosin (Kumar et al., 2014). It actively severs actin filaments as observed through fluorimetric and TEM analyses. Elucidation of the NMR model revealed the ADF-homology domain similar to EhCoactosin, however, with completely opposite biochemical activity. Critical differences in the sequence of EhActo, compared to other cofilins, unravel unique mechanisms for this family of proteins, yet to be tapped. EhActo was present in the phagocytic cups of the amoebic cells throughout the process of phagocytosis. Finally, EhActo interacts directly with EhP3 (*Entamoeba histolytica* 14-3-3 protein isoform 3), despite lacking the identified 14-3-3 binding sites. These results hint at the unexplored differential regulatory mechanisms operational in this primitive pathogen.

## Results

### Sequence Analysis Reveals the Absence of the Family Defining Serine Residue in EhActo

**FIGURE 1:**
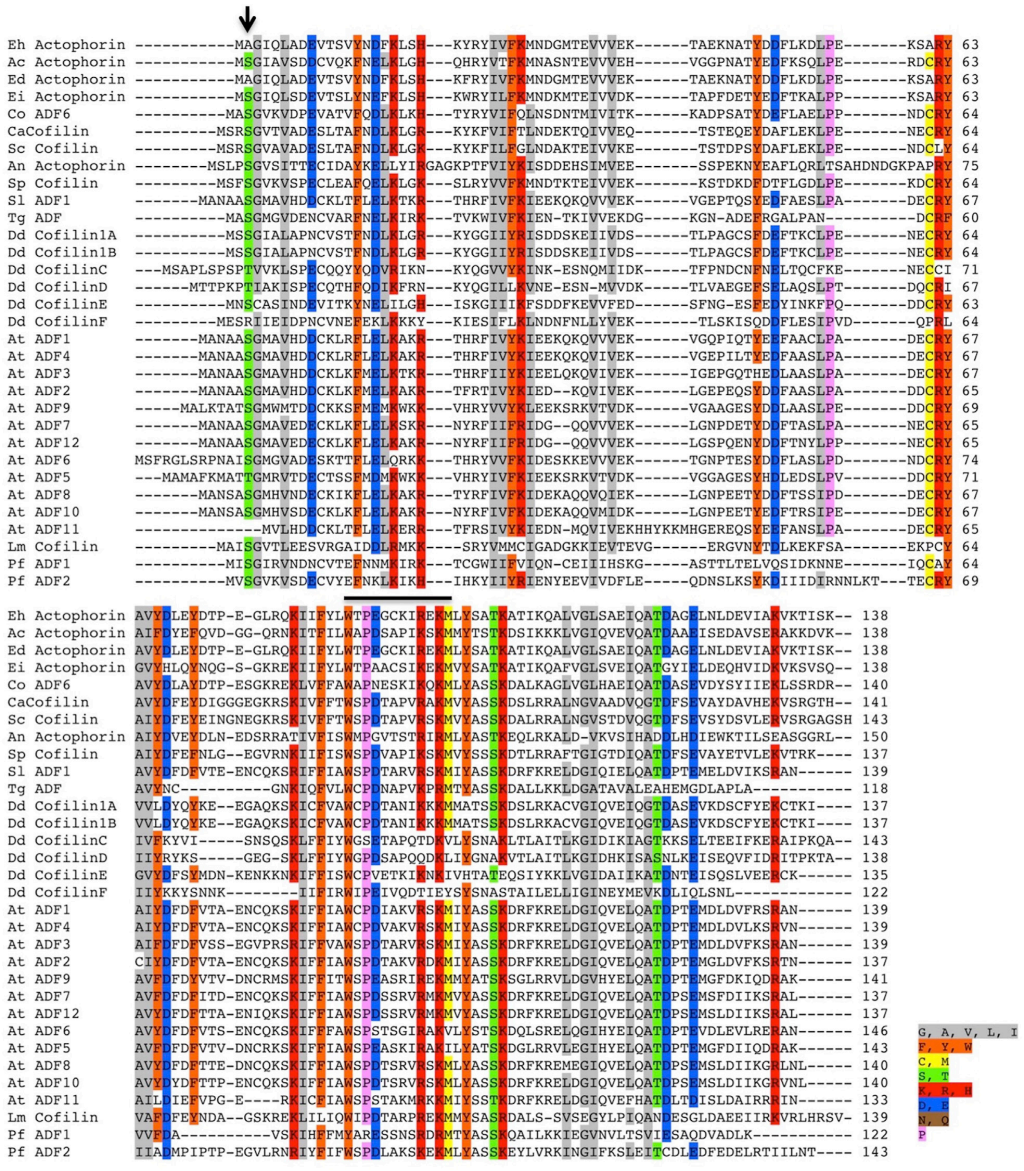
Multiple sequence alignment of several ADF/Cofilin proteins. The sequence conservation can be seen amongst several ADF/Cofilins present in different organisms. The alignment was created using the ClustalW algorithm, and the color key used to label different residues has been specified along with the image. The conserved Ser residue involved in the phospho-regulation present at the first three residues amongst various proteins is absent in EhActo, marked by the black arrowhead in the image. Abbreviations used are as follows: Eh, *Entamoeba histolytica*, Ac, *Acanthamoeba castellanii*, Ed, *Entamoeba dispar*, Ei, *Entamoeba invadens*, Co, *Capsaspora owczarzaki*, Ca, *Candida albicans*, Sc, *Saccharomyces cerevisiae*, An, *Aspergillus niger*, Sp, *Schizosaccharomyces pombe*, Sl, *Solanum lycopersicum*, Tg, *Toxoplasma gondii*, Dd, *Dictyostelium discoideum*, At, *Arabidopsis thaliana*, Lm, *Leishmania major*, Pf, *Plasmodium falciparum*. The sequences were selected based on the relatively higher sequence similarities.

Only a handful of proteins are annotated as Actophorin in the Uniprot database (UniProt, 2019), that is, the cofilin homologue characterised from *Acanthamoeba castellani* (AcActo). Other homologues are generally named ADF or cofilin in several other organisms. *Entamoeba histolytica* codes for 11 proteins with the ADF-H domain, with C4LVG4 (EHI_197480), labelled as actophorin (Rath and Gourinath, 2020). Ser1 phosphorylation of AcActo is one of the critical regulatory mechanisms; however, EhActo lacks Serine at any of the first five residues (Figure 1). This opens up exciting avenues to search for unique regulatory pathways in this pathogen. Also, EhActo neither contains an NLS or NES sequence, unlike other cofilins (Kalderon et al., 1984; Munsie et al., 2012). Thus, EhActo should not be involved in shuttling actin monomers across the nucleus.

### Tracing the Phylogeny of EhActo

In one of the previous studies, EhActo was present as the separately diverged protein branch compared to ACs from several organisms (Maciver and Hussey, 2002). EhActo shares maximum identity with ACs from fellow amoeboid protists, *Acanthamoeba castellani* and *Capsaspora owczarzaki*, followed by fungal counterparts. Surprisingly, it shares the least identity with the C-terminal ADF-H domain from EhTWF (Supplementary Figure S1). Also, where EhActo is 99% identical to putative actophorin from *E. dispar*, with *E. invadens* that percentage scrolls down to 68%. Hence, amoebic proteins continue to display a high degree of divergence from other organisms.

### EhActo Interacts With Only the Filamentous Form of Actin

**FIGURE 2:**
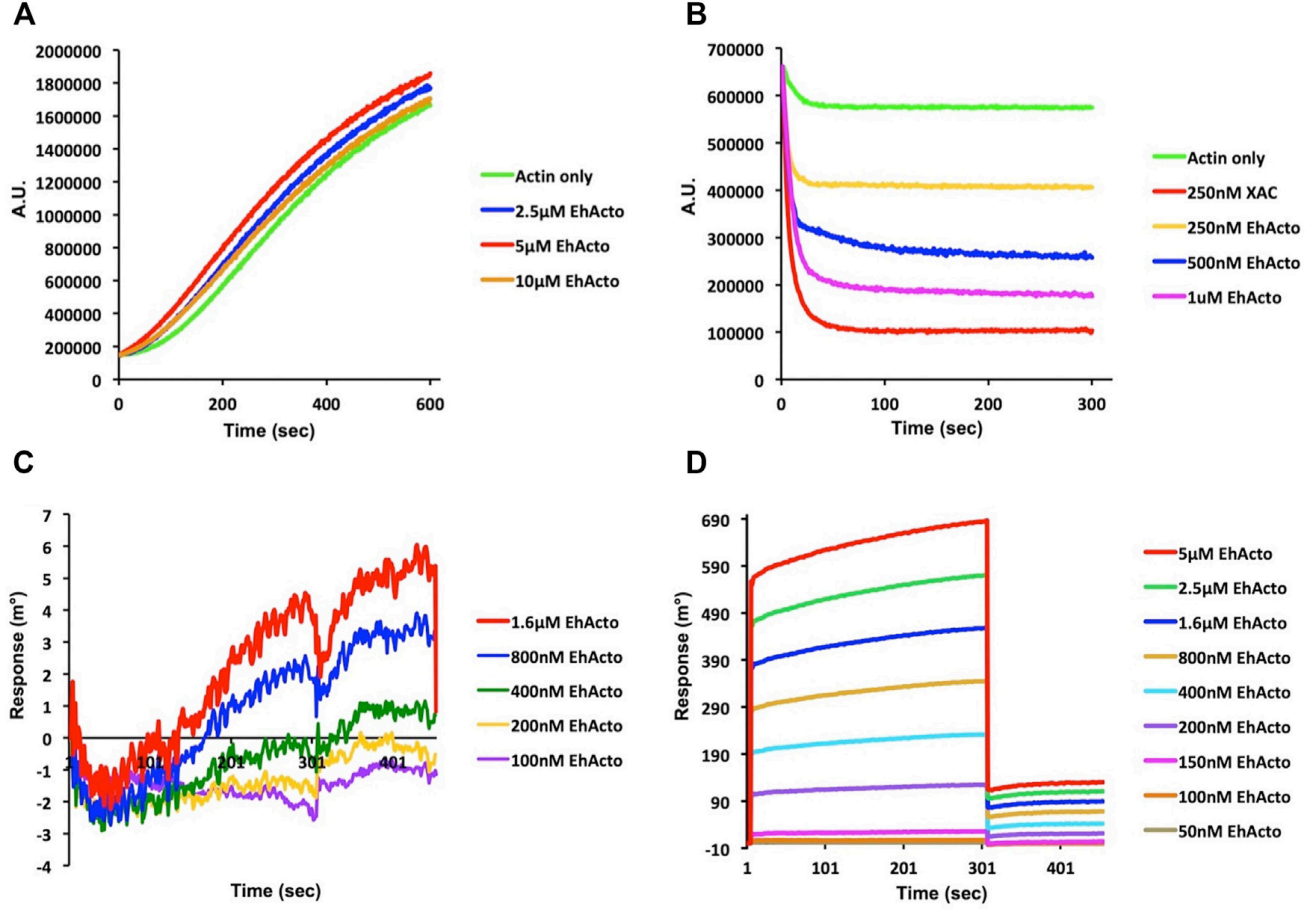
EhActo preferentially binds to actin filaments only. The actin-binding activity of EhActo was monitored qualitatively by fluorescence spectrometry **(A,B)** and quantitatively by Surface Plasmon Resonance (SPR) **(C,D)**. **(A)** 10% pyrene labelled actin monomers (4 μM) were allowed to polymerise for 10 min. EhActo was added to subsequent reactions with increasing concentrations; however, there was no change observed in the actin polymerisation. **(B)** 10% pyrene (4 μM) F-actin was allowed to self-depolymerise in the control reaction. 250 nM XAC (*Xenopus* cofilin) was taken as the positive control for actin depolymerisation. EhActo rapidly depolymerised F-actin in a concentration-dependent trend. **(C)** The SPR sensor grams obtained when G-actin was immobilised onto the chips, displayed no significant binding between the two. **(D)** Neat association and dissociation curves were observed when EhActo interacted with the immobilised F-actin molecules. The affinity constant for this interaction was calculated to be 249 nM.

As a preliminary study, we analyzed the co-sedimentation of EhActo with F-actin. EhActo pelleted down with actin filaments after the ultracentrifugation (Supplementary Figure S2). Further, on the addition of EhActo to the actin filaments, the actin band was visible in the supernatant fraction. This explains the severing by EhActo, releasing the lighter filament fragments. We used pyrene-actin fluorescence assay and surface plasmon resonance to characterize the actin-binding abilities of EhActo. Earlier reports on AcActo described both G- and F-actin binding activities for it. In the case of EhActo, we only found F-actin binding and depolymerising activity. When pyrene-actin was allowed to polymerise in the presence of increasing concentrations of EhActo, its kinetics was the same as the one for the actin alone sample (Figure 2A). Hence, EhActo does not sequester G-actin to inhibit active polymerisation. However, when F-actin was incubated with various concentrations of EhActo, sharp depolymerisation was observed, comparable to the cofilin from *Xenopus Laevis* (XAC), used as control. The depolymerisation reaction occurred within the first 30 s of the kinetic plot (Figure 2B).

The quantitative SPR experiments well ratified these qualitative observations. G-actin was immobilized on the gold chip, and different concentrations of EhActo were allowed to interact with it, but the sensograms displayed no binding between the two (Figure 2C). However, when F-actin was immobilized, a concentration-dependent binding of EhActo was observed (Figure 2D). A sharp curve was seen for association and dissociation kinetics with an overall affinity constant of 249 nM. In both the curves, within the first few seconds of the reaction, the maximal activity was observed. EhActo constantly displayed only F-actin binding through the co-sedimentation assay, spectrofluorometry, and SPR.

### Capturing EhActo in its Severing Action

**FIGURE 3:**
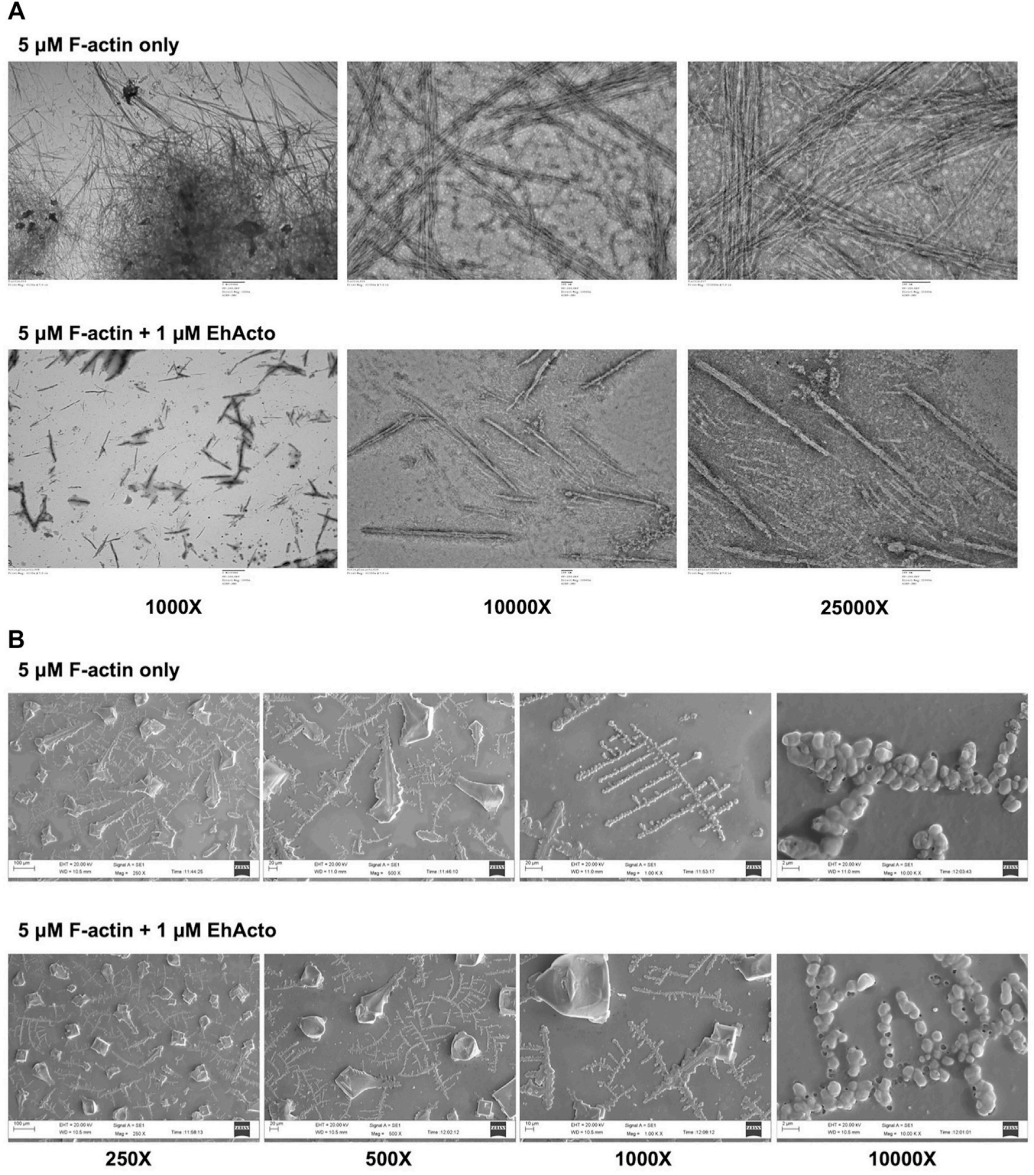
EhActo severs actin filaments. **(A)** TEM and **(B)** SEM were used to visualise EhActo in action. As hinted by cosedimentation, these electron microscopy images confirmed that EhActo severs actin filaments. 5 μM actin filaments were taken as control samples, and the same amount of F-actin was incubated with 1 μM EhActo. Shorter severed actin filament fragments can be seen in the lower panels of the image series [in both **(A,B)**].

Fluorescence spectrometric analysis points towards the depolymerising activity of EhActo. We used TEM and SEM to visualise the same in action. In order to do so, we incubated F-actin for a few seconds before loading the sample onto the grid. F-actin alone formed long filaments, as shown in Figure 3A. When 1 μM EhActo was allowed to act on these polymers, it produced severed actin filaments using a chopping mechanism. This severing action is comparable to AcActo, the other amoebic homologue (Maciver et al., 1991b).

Similarly, when we visualised SEM images, long filaments of actin were present in the control sample, and (1 μM) EhActo drastically reduced the filament lengths as discernible at ×1,000 magnification (Figure 3B). An even closer look at ×10,000 magnification captured the severed actin filaments lying close to each other. Hence, EM studies corroborated the SPR and fluorescence studies, revealing the severing mechanism of EhActo. This activity is similar to other studies of actophorin/ADF proteins.

### Structural Details of EhActo Revealed by NMR Studies

**FIGURE 4:**
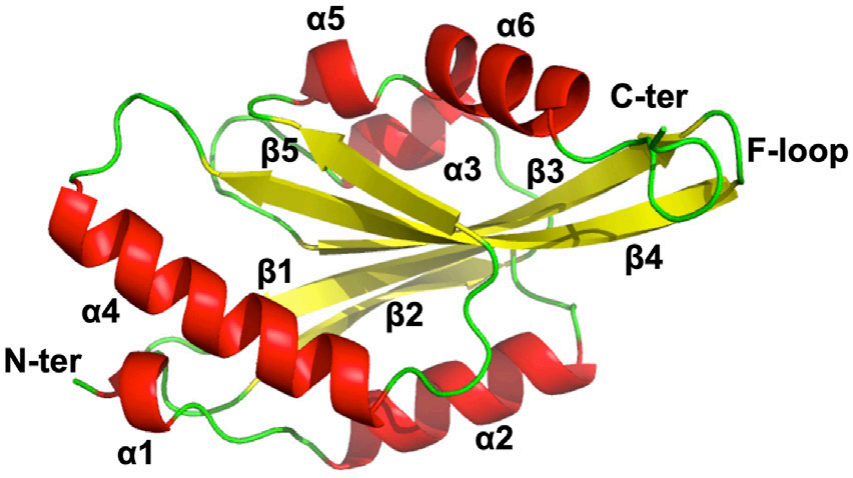
NMR model of EhActo reveals structural conservation of ADF/Cofilin family. NMR studies determined the chemical shift derived solution structure model of EhActo using CS-ROSETTA. A core of β-sheets (Yellow) can be seen caged by six α-helices (Red). Random coils (Green) connect these secondary structures. The N- and C-terminals were diagonally opposite to each other.

Several ADF/cofilin structures are now available in PDB for analysis; however, for actophorin, only two structures are present from *Acanthamoeba* species. NMR studies unravelled EhActo structural details, and we found that it contains four α helices and two small helices caging five antiparallel β sheets. This is a typical secondary structure pattern of the AC family of proteins where a central core of β-sheet is surrounded by α-helices. α1 (2–5) and α5 (123–126) are single half-turn helices, while α4 (94–110) is the longest one. α2 (10–22) and α3 (51–57) parallel each other in the plane. β1 (26–32) and β3 (65–74) are parallel to each other and are antiparallel to β2 (39–46), β4 (77–88), and β5 (116–120). It also flaunts the family defining F-loop that is involved in F-actin binding. α6 (128–138) is present very close to F-loop; thus, it could provide additional contact points for F-actin. The N-terminal and C-terminal ends lie diagonally opposite to each other (Figure 4).

### Structural Homologues of EhActo Display Differences

**FIGURE 5:**
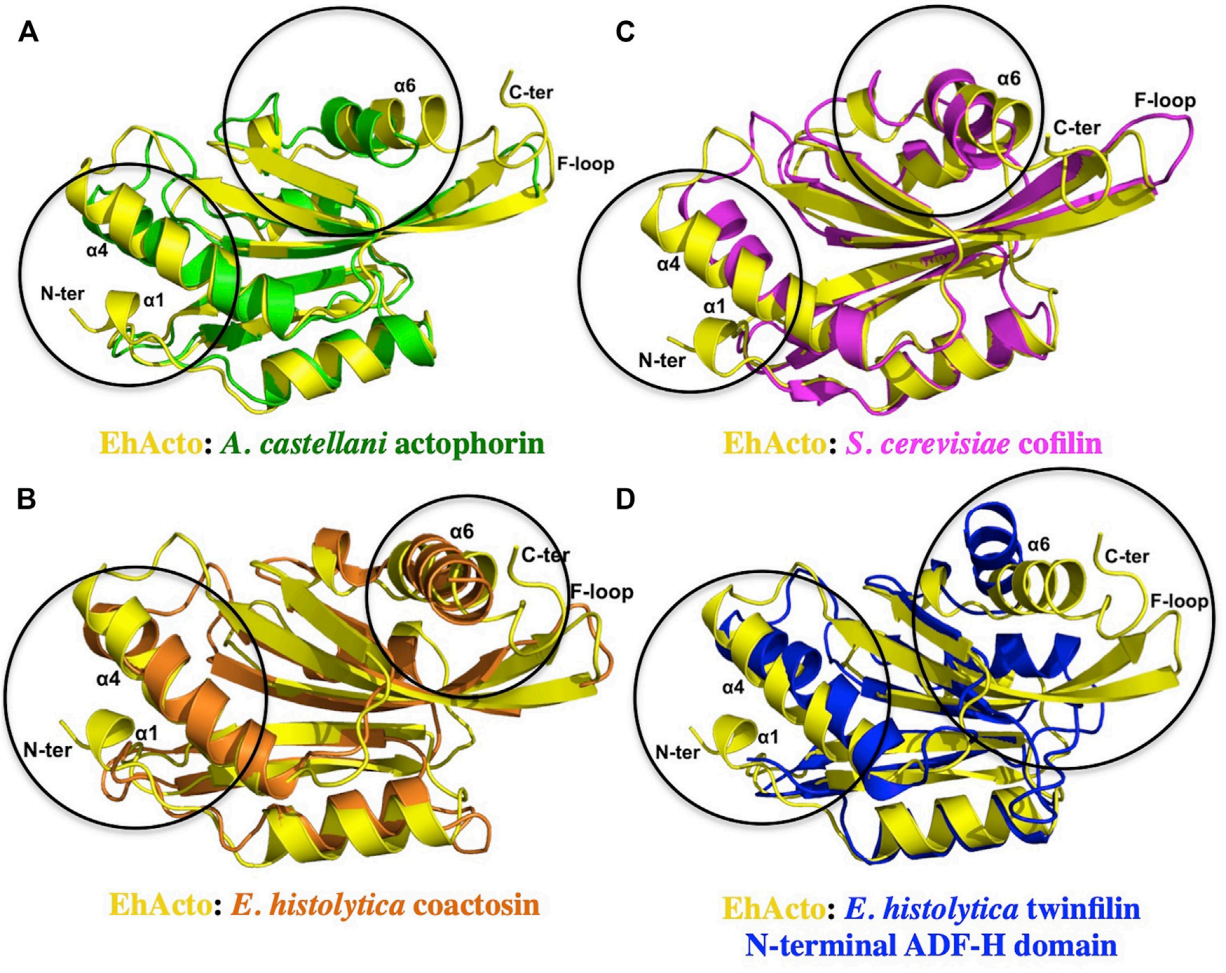
Structural conservation amongst different ADF/Cofilins. A single structure of actophorin is available in the PDB form **(A)** *Acanthamoeba castellani* (Green). Along with that, we compared the structure of EhActo (Yellow) with **(B)** EhCoactosin (Orange) and **(D)** NT EhTWF (Blue), the other two ADF-H domain-containing proteins from *Entamoeba histolytica*. **(C)** Yeast cofilin (Magenta) is one of the well-studied proteins, and so we also used it in our analyses. Except for NT EhTWF, all the other proteins had the F-loop that is a signature of this family of proteins. Overall, the structural features were found to be conserved amongst all of them.

We compared EhActo model with other ACs from *Entamoeba histolytica*, itself since they share the least sequence homology. *Acanthamoeba castellanii* (AcActo) and *Saccharomyces cerevisiae* (ScCof) cofilins were also analyzed for their structural variations to correlate with functional altercations. AcActo (PDB ID: 1AHQ) is currently the sole Actophorin with a PDB available in the database. With an alignment of 133 residues, we obtained an RMSD of 2.1 Å between AcActo and EhActo. A comparison with yeast Cofilin (ScCof) (PDB ID: 1CFY), EhCoactosin (PDB ID: 4LIZ), and NT EhTWF (PDB ID: 6K2F) yielded RMSD values of 2.1, 2.6, and 2.7 Å, respectively (Fedorov et al., 1997; Leonard et al., 1997; Kumar et al., 2014). Few marked variations were visible on comparison; the N-terminal end was longer in EhActo with an added small helix (Ala2 to Gly5). β2 of AcActo was very small with respect to β2 of EhActo (Figure 5A). The most extended helix (α4) was bent in all structures except EhActo, where it was more straightened out. The angular variation is maximum with AcActo and ScCof and minimum with NT EhTWF (Figure 5D). There is no helix corresponding to EhActo α5 in ScCof and AcActo. The last helix was longer and tilted away from the core in EhActo. ScCof has an additional β sheet at its N-terminal, unlike any other protein (Figure 5B). The distorted last helix of EhCoactosin encompasses both α5 and α6 of EhActo (Figure 5C). Also, β3 and β4 are the shortest in EhCoactosin, while α2 is the longest in NT EhTWF. Finally, NT EhTWF has two significant differences: the C-terminal α helix is bent inwards, lacks the F-loop, and is present in all other homologues.

The minor differences observed in the structure explains some of the functional variations. As mentioned earlier, unlike all other homologues, EhActo lacks monomeric G-actin binding. The critical secondary and tertiary structural changes like longer N-terminal terminal end with additional small helix and the orientation of the last helix could be asserting the altered functional role for EhActo, since these fall in the actin-binding interface.

### Unique Surface Charge Distribution Pattern of EhActo

AC family has a unique surface charge signature, with one face of positive residues, while the opposite faces negatives. ScCof was an ideal example for the same (Supplementary Figure S3A). EhCoactosin was surprisingly different, with both surfaces being negative (Supplementary Figure S3B). NT EhTWF has the third variation of major hydrophobic regions covering the entire structure (Supplementary Figure S3C). AcActo follows the typical AC pattern of surface charge distribution; however, the α3 is positively charged on both faces (Supplementary Figure S3D). α3, along with positively charged C-terminal, have been implicated as actin-binding region (Fedorov et al., 1997; Leonard et al., 1997). In the case of EhActo, these particular regions tell a different story (Supplementary Figure S3E). The C-terminal end is relatively more negatively charged, while α3 carries a lesser positive charge with hydrophobicity. The loop before the C-terminal helix and the helix itself is known to bind actin molecules (Paavilainen et al., 2008). Another major point for consideration is that the F-loop in EhActo is entirely negative with intermittent hydrophobic residues, while in AcActo, it is negative on one face and positive on the other.

The surface charge distribution observed in EhActo is markedly different from other homologues. Typically, AC family proteins project one negative and one positive surface, as seen in ScCof. However, AC family proteins from *E. histolytica* tend to break the norm; EhCoactosin is mainly negative, while NT EhTWF displays more number of hydrophobic regions. Similarly, EhActo contains both negative and hydrophobic surface areas. Except for EhActo, all the other homologues bind both forms of actin. On the other hand, EhCoactosin and NT EhTWF stabilize actin filaments rather than depolymerising them.

### EhActo Localises Near Membrane in *Entamoeba histolytica*

**FIGURE 6:**
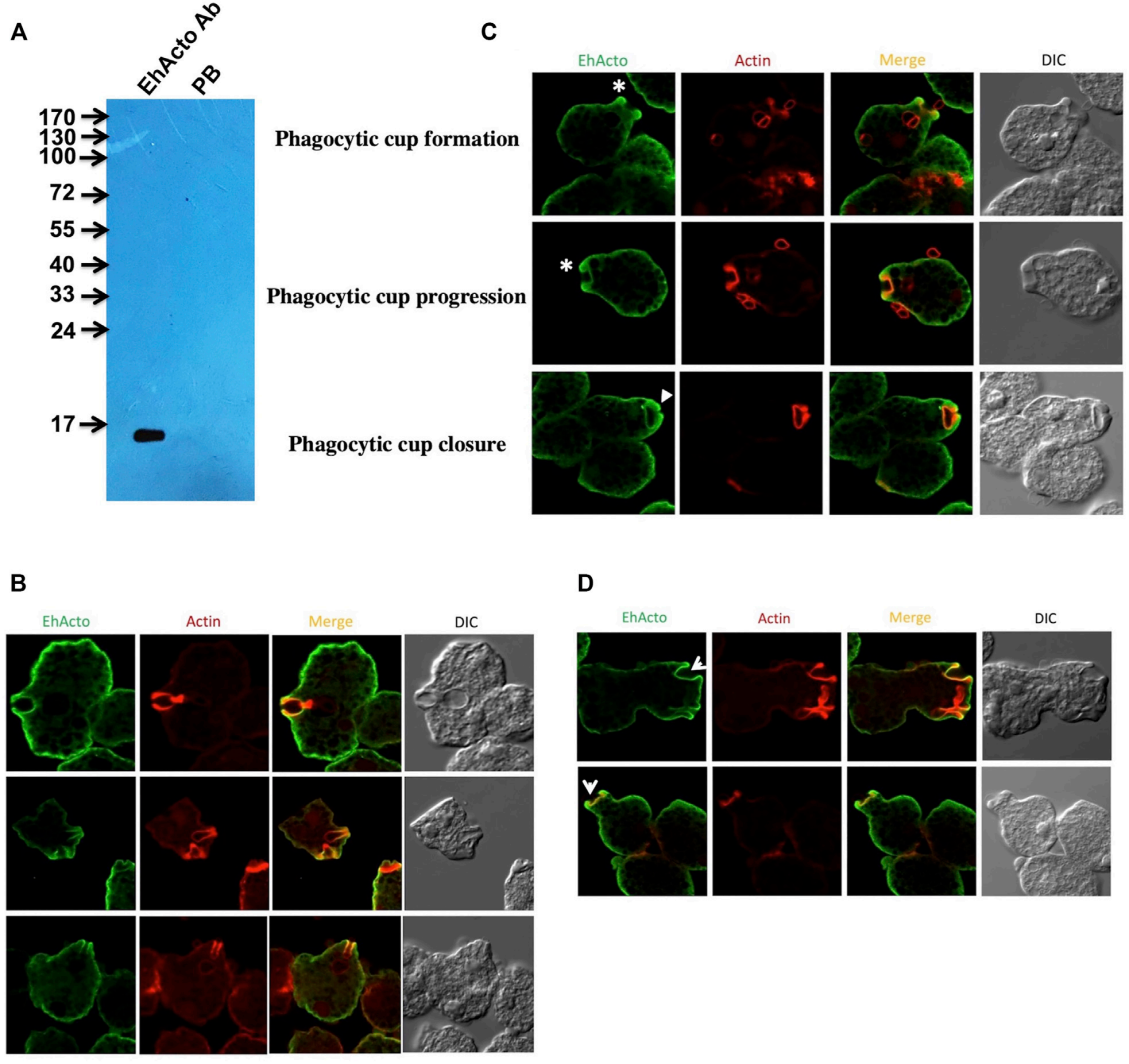
Cellular localisation of EhActo in the amoebic trophozoites. **(A)** Western blot analysis was used to check the specificity of the EhActo antibody raised against the purified protein. *Entamoeba* cell lysate (80 μg) was probed with anti-EhActo (1:3,000) and Pre-bleed (PB) was taken as control. The expected size of EhActo is ∼16 kDa. Confocal microscopy revealed the presence of EhActo lining the membrane of the amoeba. **(B)** EhActo (Alexa-488) colocalised with actin stained with TRITC-phalloidin. **(C)** The stars mark the presence of EhActo all along the process of erythrophaocytosis, from the beginning of the cup formation till its closure. (Scale bar is 10 μm). **(D)** EhActo was present in the pseudopods thrown by the amoebic trophozoites; marked by arrowheads.

We performed immunofluorescence-staining studies using antibodies raised against purified recombinant EhActo (Figure 6A). EhActo was observed to be concentrated near the plasma membrane as visualized by confocal microscopy (Figure 6B). However, it was ubiquitously present throughout the cytoplasm as well. The localisation of EhActo was seen parallel with actin all along with the pseudopod formation (Figure 6C). EhActo was present at the tips of the actin-rich phagocytic cups during the presence of red blood cells, during erythrophagocytosis. The protein was constantly present till the closure of the phagocytic cups (Figure 6D). Hence, the involvement of EhActo is to regulate the basal level of actin dynamics, in general, near the membrane of this pathogen. The presence of EhActo at the site of pseudopod formation is critically vital for the rapid actin turnover required during motility.

Earlier studied amoebic proteins like EhCoactosin, EhARPC1 and EhARPC2, EhMyosin1b, EhP3, EhLIMA, EhRho1, EhNCABP166, enter and exit the phagocytic cup formation site at different stages (Voigt et al., 1999; Wender et al., 2007; Campos-Parra et al., 2010; Kumar et al., 2014; Babuta et al., 2015; Babuta et al., 2018; Bharadwaj et al., 2018; Agarwal et al., 2019; Gautam et al., 2019). However, the omnipresence of EhActo throughout all the cellular processes like motility and erythrophagocytosis explains its critical importance in amoebic biology.

### EhActo Induces Conformational Changes in F-Actin

**FIGURE 7:**
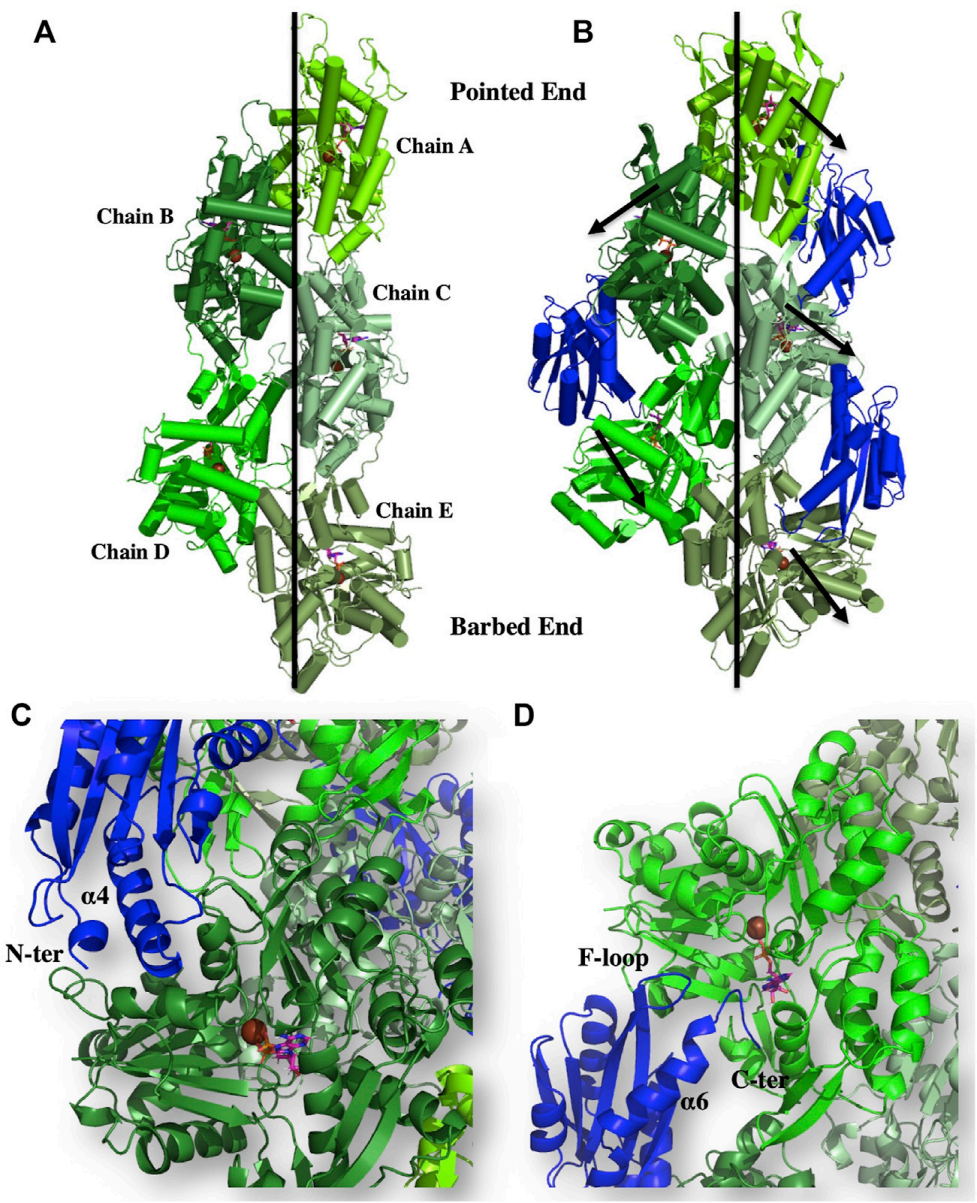
Binding of EhActo to actin filaments, as explained through modelling studies. The cartoon representation shows the proposed binding mode of EhActo (Royal) with the actin filament (Different shades of Green). **(A)** The actin filament (PDB ID: 5ONV). **(B)** EhActo decorated actin filament was modelled based on the PDB 5YU8. The black arrows indicate the outward expansion of the structure upon EhActo binding. **(C)** A closer look at the interacting region between one of the actin monomer and EhActo while **(D)** represents the interface of the same EhActo molecule with the other actin monomer. The N-terminal, α4, α6, F-loop, and the C-terminal have been labelled as the interacting zones.

As stated above, our biochemical data identified that EhActo binds and severs F-actin only. The electron microscopy data clearly displayed the severing action; therefore, we superimposed EhActo onto the cofilin bound F-actin structure (PDB ID: 5YU8) to understand the binding mechanism. A comparison between unbound (PDB ID: 7BT7) (Figure 7A) and EhActo bound F-actin displayed structural changes that explain the action of EhActo. Both the structures superimposed with an RMSD of 2.7 Å. As visible in Figure 7B, the binding of EhActo resulted in a more open conformation for F-actin, thus loosening the polymer. There was significant angular and translational displacement between the two structures. All the actin molecules displaced outwards, away from the central axis (arrow marked in Figure 7B). These parameters are tabulated to show the relative movement of the monomers (Table 1). When we zoom in to look at the EhActo-F-actin interface, we can see two primary contact points. A single molecule of EhActo contacts two actin molecules. On one end, the interactions are mediated by F-loop and the C-terminal helix (α6) (Figure 7D), while the N-terminal end and α4 contact the second actin molecule (Figure 7C). These interactions are very similar to other reported cofilins. These interacting regions are oriented differently when compared to other homologues, thus explaining the functional distinction. The direction of α6 is significant in mediating actin contacts in the case of EhActo.

**TABLE 1:** The angular and linear displacement observed between unbound actin molecules in its filament form and EhActo-bound actin molecules in the filaments as shown in Figures 7A,B.

Monomer	Angular difference (°)	Displacement (Å)
Chain A	12.03	2.96
Chain B	8.97	1.97
Chain C	5.37	1.21
Chain D	6.48	2.34
Chain E	9.37	1.60

### EhP3 (14-3-3) Interacts Directly With EhActo

**FIGURE 8:**
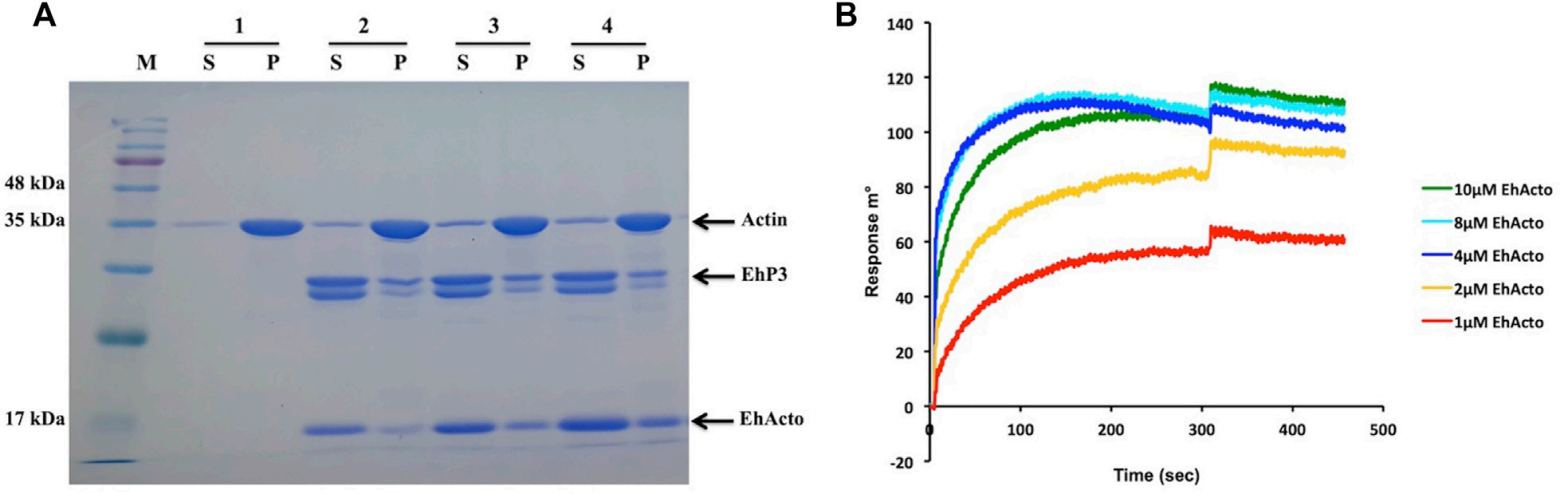
EhP3 interacts with actin *via* ADF/Cofilins. **(A)** F-actin cosedimentation assay was performed with EhActo and EhP3. With the addition of EhActo, there was an increase of EhP3 in the pellet fractions along with F-actin. Actin and EhP3 were maintained at a constant concentration of 5 μM in all the reactions. Sample 1 is actin only control; sample 2, 2.5 μM EhActo; sample 3, 5 μM EhActo; and sample 4, 10 μM EhActo. **(B)** These findings were corroborated by SPR studies, where EhP3 was immobilised on the sensor chips while the increasing concentration of EhActo were passed in the running buffer to determine the binding affinity. The affinity constant for EhActo was 1 μM.

Three recognition motifs have been characterised in 14-3-3 binding partners that contain phosphoSer/Thr (**p**S/**p**T) (Bridges and Moorhead, 2005). Motif I: (RSX**p**S/**p**TXP), Motif II: (RXY/FX**p**S/**p**TXP), and Motif III: **p**S/**p**TX1-2-COOH, where X is any amino acid except Proline. However, a couple of other sequence motifs were also located on the non-phosphorylated substrates: GHSL and WLDLE (Ferl et al., 2002). The discovery of these new binding sites presents broader roles for 14-3-3 proteins than just being phosho-sensors. Surprisingly, in the case of EhActo, the sequence analysis revealed that none of these motif signatures is present in it. A recent article by Agarwal et al. (2019) described the presence of three 14-3-3 isoforms (namely EhP1, EhP2, and EhP3) in *Entamoeba histolytica*. The work reported EhCoactosin’s interaction with EhP3, despite the lack of a consensus 14-3-3 binding site, we were intrigued to analyze the same for EhActo (Agarwal et al., 2019). It is already published that EhP3 does not interact with actin (Agarwal et al., 2019) and (Supplementary Figure S4); thus, for qualitative assessment, we deployed a co-sedimentation assay, where we incubated F-actin with EhP3 and EhActo. The presence of EhActo increased the presence of EhP3 in the pellet fraction (Figure 8A). Thus, EhActo probably bridges a connection between EhP3 and actin, similar to EhCoactosin. Next, we used SPR interaction kinetics to validate the direct protein-protein interaction (Rath et al., 2020). We found that EhActo binds to EhP3 with an affinity constant of 1 μM (Figure 8B).

EhP3 controls the recruitment of EhCoactosin at the phagocytic cups, wherein the decrease in the cellular concentration of EhP3 delayed phagocytosis. Thus, EhP3 could be deploying a similar carrier function for EhActo, although this awaits further validation.

## Discussion

Discovered as the amoebic homologue of Cofilin, actophorin has since displayed all the characteristics of the ADF/Cofilin family proteins. The protein was earlier studied in *Acanthamoeba*, the soil-dwelling protist. We have extended the study to understand the same protein in *Entamoeba histolytica*, the enteric protozoan globally infecting humans. Although both the proteins are amoebic homologues, they share just 47% sequence identity. The most distinctive feature for EhActo is the absence of the regulatory Serine residue at its N-terminal. Ser1 (AcActo) or Ser3 (mammalian Cofilin) are subjected to phosphocycling as a switch to regulate it. This opens the possibility of a different mechanism operational in *Entamoeba histolytica* for the actophorin regulation.

One of the key findings of our study was the inability of EhActo to interact with G-actin, as shown by pyrenyl-actin fluorescence and SPR studies. Unlike AcActo, EhActo interacted with only the filament form of actin. A Cofilin peptide WAPESAPLKSKM was reported to be the site of G-actin interaction (Yonezawa et al., 1991). However, in EhActo, there are significant substitutions at this region with the following sequence, WTPEGCKIREKM (Figure 1). These residue changes could explain the lack of G-actin binding by EhActo. In the co-sedimentation assay, we found actin in the supernatant fraction upon addition of EhActo that hinted at the depolymerising/severing action. Fluorescence studies revealed the fast kinetics of the depolymerisation activity and visualization through TEM, and SEM further confirmed the severing action of EhActo.

We determined the model of EhActo using NMR spectroscopy, and the model was typical of the AC family, with few variations. Our model of EhActo-F-actin projects the structural changes induced upon the binding of EhActo. The overall F-actin broadens outwards from the central axis. The F-loop, α4, α6, and the N-terminal are the contact regions for two adjacent actin monomers in the filament.

EhActo co-localized with actin in the amoebic trophozoites and lined the membrane. The location of EhActo at the membranes can be explained by the presence of the conserved basic amino acid residues at positions 93 and 95 that are known to bind PIP_2_ present in the membrane (Van Troys et al., 2000). Thus, EhActo probably associates with PIP_2_ all the time in the cells. It was present at the tips of pseudopods and phagocytic cups. EhActo remained at the tips till their closure during erythrophagocytosis. Our data displays cellular localization of EhActo during cellular motility and phagocytosis. The analysis reveals that cells are polarized during phagocytosis *via* different regulatory pathways recruiting certain proteins at phagocytic site. EhActo probably aids in actin monomer recycling by rapid depolymerization. This activity is crucial during the formation of phagosomes, as the pseudopods need to first progress in order to capture their prey and then regress to engulf it.

Direct SPR studies revealed association of EhP3 with EhActo at concentrations above 1 μM. These results were surprisingly different from the predictions. Firstly, EhActo does not contain any consensus 14-3-3 binding motifs, yet it interacts with good affinity. Secondly, EhActo lacks the Ser1 (counterpart of mammalian Ser3) residue whose phosphorylation is essential for the 14-3-3 binding mechanism. Since our studies used proteins purified from the *E. coli* cells, they most probably do not carry any posttranslational modifications like phosphorylation. Hence, the two earlier described necessary conditions, phosphorylated Serine and 14-3-3 recognition motifs are rendered dispensable in amoebic EhActo. Thus, in conclusion, the discovery of an alternate binding mechanism for 14-3-3 can be pencilled in, and, therefore, further studies are required to understand these potential differences.

Thus, in conclusion, we report the first cofilin from *Entamoeba histolytica*. The earlier reported AC family protein EhCoactosin failed to depolymerise/sever actin filaments (Kumar et al., 2014). Functionally, EhActo behaves similar to the EhCoactosin ΔF mutant that lacks the actin-binding F-loop. However, EhActo is fully capable of severing the actin filaments and works in actin regulation during basic processes in *Entamoeba histolytica* trophozoites. Presence of EhActo at phagocytic cup formation explains its function of active depolymerisation required for rapid actin dynamics during phagocytosis. Hence, involvement of EhActo during its virulence factor describes it as the single cofilin present aiding the amoebic pathogenesis via phagocytosis.

## Materials and Methods

### Cloning and Purification of EhActo

The gene corresponding to the amoebaDB ID: EHI_197480 was annotated as actophorin, hereafter referred as EhActo. It was cloned in pET28 (b) vector between the *Nco*I and *Xho*I restriction sites. The clone was overexpressed in BL21 (DE3) cells for the generation of recombinant protein with a C-terminal His_6_ tag. Ni-NTA chromatography was used as the first step of the purification in a buffer with 100 mM Tris pH 8.0, 0.1 mM EDTA, and 5 mM β-Mercaptoethanol. The protein was eluted in a buffer with 100 mM Imidazole. The second step Gel filtration chromatographic purification was performed in G75 superdex column (GE Healthcare). The protein eluted as a monomer and was concentrated in a Millipore centricon of 5 kDa cutoff.

### Sequence and Phylogenetic Analyses

All the homologous sequences were extracted from the UniProt database (UniProt, 2019) and were aligned by ClustalW in the MEGA 7 software suite. The resultant alignment was used to generate the phylogenetic tree by the Maximum Likelihood method based on the Jones-Taylor-Thornton model (Kumar et al., 2016). 500 bootstrap replicates were deployed to generate phylogenetic tree. The tree with the maximum score was selected and the final figure was prepared by iTOL (Letunic and Bork, 2019). All the sequences used have been tabulated and provided as Supplementary Table S1.

### Protein Preparation for NMR Spectroscopy

For the preparation of isotopic labeled *U*-^15^N or *U*-^13^C,^15^N—labeled recombinant protein, 2.5 g L^−1^ ^13^C_6_ - D-glucose and 1.0 g L^−1 15^NH_4_Cl (Cambridge Isotope Laboratories), as sole carbon and nitrogen sources, respectively, in M9 minimal media, yielding the uniformly ^13^C,^15^N- labeled protein. The NMR buffer used for final protein preparations comprised 10 mM sodium phosphate pH 7.0, 1.8 mM potassium phosphate, 60 mM NaCl, 2.7 mM KCl and 5% D_2_O.

### Actin Filament Depolymerisation Assay

10% pyrene labelled G-actin at a concentration of 10 μM was polymerised to F-actin for one and a half hour in the F-buffer (10 mM Tris-Cl pH 8.0, 0.2 mM DTT, 0.7 mM ATP, 50 mM KCl, and 2.0 mM MgCl_2_). Thus formed F-actin was subjected to depolymerisation kinetics. 4 μl of actin in 66 μl F-buffer was taken in the quartz cuvette in a reaction volume of 80 μl completed with HKG5 buffer (20 mM HEPES pH 7.5, 50 mM KCl, and 5% Glycerol) or EhActo. QM 40 PTI NJ fluorimeter was used to monitor N-pyrene fluorescence (excitation wavelength at 365 nm, emission wavelength at 407 nm) for 600 s. We used *Xenopus* cofilin1 (Xac1), a known actin-depolymerising factor, as the positive control (Rosenblatt et al., 1997).

### Actin Co-Sedimentation Assay

5 µM rabbit muscle G-actin was polymerised in F-Buffer (10 mM Tris-Cl pH 8.0, 0.2 mM DTT, 0.5 mM ATP, 50 mM KCl, 2 mM MgCl_2_) for 1.5 h at room temperature. 5 µM of EhActo (or F-Buffer) was added to the hence formed F-actin and the reaction volume was made up to 150 µl. Reaction mixture was incubated for 30 min at room temperature. The samples were then ultra-centrifuged at 100,000 g for 45 min at 4°C. After centrifugation, the supernatant was pipetted out and the pellet fractions were reconstituted in F-Buffer. These were analyzed on a 12% SDS-PAGE after the Coomassie blue staining. In the case of EhP3 experiments, both actin and EhP3 were used at 5 µM concentration, with variable EhActo concentrations.

Similarly, G-actin binding was also analyzed as well. 5 µM rabbit muscle G-actin in G-buffer (10 mM Tris-Cl pH8.0, 0.2 mM DTT, 0.5 mM ATP) was either incubated alone or with EhActo (and EhP3) half an hour at room temperature. Ultra-centrifugation was performed at 100,000 g for 45 min at 4°C. Supernatant and pellet fractions were separately collected and analyzed on 12% SDS-PAGE post Coomassie blue staining.

### Surface Plasmon Resonance

SPR studies using Autolab ESPIRIT were used to determine the binding affinities between various protein molecules. Here, we followed the general protocol (Rath et al., 2020), where, G-actin, F-actin, or EhP3 (*E. histolytica* 14-3-3 protein isoform 3) were immobilised (separately on three different chips) to the activated sensor surface at a concentration of 10 µM in filtered (0.22 µm pore size) and degassed 10 mM sodium acetate buffer (pH 4.5). The chip surface was blocked using 100 mM ethanolamine at pH 8.5. Regeneration was carried out by 50 mM NaOH. The running buffer used for the experiments were: G-actin running buffer: 10 mM HEPES, 0.2 mM Calcium Acetate, 0.5 mM DTT, 0.2 mM ATP and 0.005% Triton-X100, F-actin running buffer: 10 mM HEPES, 150 mM KCl, 1 mM MgCl_2_ and 0.005% Triton-X100, EhP3 running buffer: 10 mM HEPES, 0.2 mM Calcium Acetate, 0.005% Triton-X100. The association kinetics (300 s) and subsequent dissociation kinetics (150 s) were recorded. Different concentrations of EhActo were used to obtain the KD values. The data collection performed at 25°C and Autolab SPR Kinetic Evaluation software was used for data analysis.

### Solution-State NMR Spectroscopy

Samples of 0.5 mM Actophorin protein was prepared in the buffer containing 10 mM sodium phosphate pH 7.0, 1.8 mM potassium phosphate, 60 mM NaCl, 2.7 mM KCl and 5% D_2_O. Bruker Avance III spectrometer equipped with a 5 mm cryogenic triple resonance TCI probe was used for the acquisition of the NMR experiments at the field strength of 500.15 MHz. Temperature used for acquisition of all the experiments was 298 K. For the assignment of protein resonances, experiments used were double and triple resonance experiments (Bax and Grzesiek, 1993), that is, ^2^D (^15^N,^1^H) HSQC, ^2^D (^13^C,^1^H) HSQC (aliphatic and aromatic), 3D HNCA, 3D HNCACB, 3D CBCAcoNH, 3D HNCO; for backbone resonance assignment, and 3D HcccoNH, 3D hCccoNH; for side chain resonance assignment. The spectra were processed using Topspin 3.1 (Bruker AG). Computer Aided Resonance Assignment (CARA) software was used for analysis and all assignments (Keller, 2004).

### Solution Structure Model Generation Using NMR Chemical Shifts

The backbone and side-chain resonances were manually assigned using Computer Aided Resonance Assignment (CARA) software. The ^1^H shifts were calibrated with respect to the 2,2-dimethyl-2-silapentane-5-sulfonate (DSS) at 298 K (0.0 ppm). Indirect referencing of ^13^C and ^15^N shifts was done indirectly in all the spectra with respect to the DSS methyl proton resonance at 0 ppm. The conformational information derived from the assignments was used for the calculation of the 3D structure model using the CS-ROSETTA software (Shen et al., 2008). 2D [^15^N, ^1^H] HSQC of EhActo is provided in Supplementary Figure S5. The NMR assignment has been uploaded to the Biological Magnetic Resonance Bank (BMRB 51258). We have further added the Cα secondary Chemical shift graph as Supplementary Figure S6.

### Superimposition of the Structural Homologues and Surface Charge Analyses

Structural comparisons were conducted with the earlier characterised ADF-H domain containing proteins from *Entamoeba histolytica*, namely, EhCoactosin (PDB: 4LIZ), NT EhTWF (PDB: 6K2F). Also, we procured PDBs for yeast Cofilin (PDB: 1CFY) and *Acanthamoeba* Actophorin (PDB: 1AHQ). A one on one superimposition was performed with the RAPIDO server (Mosca and Schneider, 2008). For the surface charge distribution, the ABPS plugin was used in the Pymol software, with a range of -5 (Red) to +5 (Blue). The images were prepared with the Pymol software (DeLano, 2002).

### Modelling of EhActo-F-Actin

Electron microscopy derived model of cofilin decorated F-actin (5YU8) (Tanaka et al., 2018) was used as the template to superimpose EhActo onto the cofilin present in the structure. 132 residues aligned with a RMSD of 2.41 Å using the RAPIDO sever (Mosca and Schneider, 2008). This final model with three EhActo and five actin molecules was subjected to energy minimization cycles using YASARA server (Krieger and Vriend, 2014). Finally, Pymol was used to create the figures (DeLano, 2002).

### Transmission Electron Microscopy

The actin filaments were viewed in a Transmission Electron Microscope (JEOL JEM-2100F) with 200 kV accelerating voltage. Control reaction was setup with 5 μM F-Actin alone. For experiments with EhActo, 5 μM F-Actin was incubated with it for a couple of minutes. The samples were then adsorbed onto carbon coated copper 400 mesh grids and extra fluid was removed after 2 min of adsorption. Grids were negatively stained using 1% uranyl acetate. Images were captured at ×10,000, ×25,000, and ×50,000.

### Scanning Electron Microscopy

Proteins are generally not visualized using SEM, however, since actin filaments are high molecular weight protein polymers, we tried to look at them through SEM. 5 μM F-Actin was used as control sample, while 5 μM F-Actin incubated with 250 nM EhActo was setup for experimental sample. A very thin film of gold and palladium (Au–Pd) was deposited on the surface of the samples to make them electrically conductive using the vacuum coating unit. This extremely fine coating was done through the evaporation of Au–Pd plate under inert atmosphere (argon environment). These samples were mounted on electron microprobe stubs. The SEM analyses were carried out with the help of a computer-controlled field emission SEM (JEOL JSM-6330F, JEOL Ltd., Akishima, Tokyo, Japan). The samples were viewed at ×250, ×500, ×1,000, and ×10,000 magnifications.

### Growth and Maintenance of Parasite *Entamoeba histolytica* Strain HM1-IMSS

The standard protocol as described in several earlier publications was followed for the growth and maintenance of *E. histolytica* strain HM-1: IMSS (Kumar et al., 2014). TYI-S-33 medium was used with 125 μl of 250 U ml^−1^ benzyl penicillin and 0.25 mg ml^−1^ streptomycin per 100 ml of medium.

### Immunofluorescence Staining

Immunofluorescence staining protocol was followed as described previously (Kumar et al., 2014). Briefly *E. histolytica* cells were resuspended in the TYI-33 medium and were then transferred and allowed to adhere on the acetone-cleaned coverslips for 10 min at 35.5°C. After the removal of the medium, the cells were fixed with 3.7% pre-warmed paraformaldehyde (PFA) for 30 min. The fixed cells were treated with 0.1% Triton X-100/PBS for 1 min for permeabilisation. This step was omitted for non-permeabilized cells. After a PBS wash, quenching was performed for 30 min with 50 mM NH_4_Cl in PBS. 1% BSA/PBS was used for blocking the coverslips for 30 min. The primary antibody was then added followed by an incubation at 37°C for 1 h. Before incubation with the secondary antibody, the cover slips were washed with PBS followed by another blocking step with 1% BSA/PBS of 30 min at 37°C. Antibody dilutions used were: Anti-EhActo at 1:3,000, TRITC-Phalloidin at 1:250 and anti-rabbit Alexa 488 (Molecular Probes) at 1:300. The samples prepared were washed with PBS again before mounting on a glass slide using DABCO (1,4-diazbicyclo (2,2,2) octane (Sigma) 10 mg ml^−1^ in 80% glycerol). Nail polish was applied to seal the edges of the coverslip. Confocal images were collected and viewed using an Olympus FluoView FV1000 laser-scanning microscope.

## Data Availability

The datasets presented in this study can be found in online repositories. The names of the repository/repositories and accession number(s) can be found below: Entamoeba histolytica Actophorin (EhActo) has Uniprot ID C4LVG4.
