# Supplementary figures and images for: Unravelling the Biology of EhActo as the First Cofilin From Entamoeba histolytica
Source: Front Cell Dev Biol. 2022 Feb 25;10:785680. doi: 10.3389/fcell.2022.785680 (PMC8914023; doi:10.3389/fcell.2022.785680)

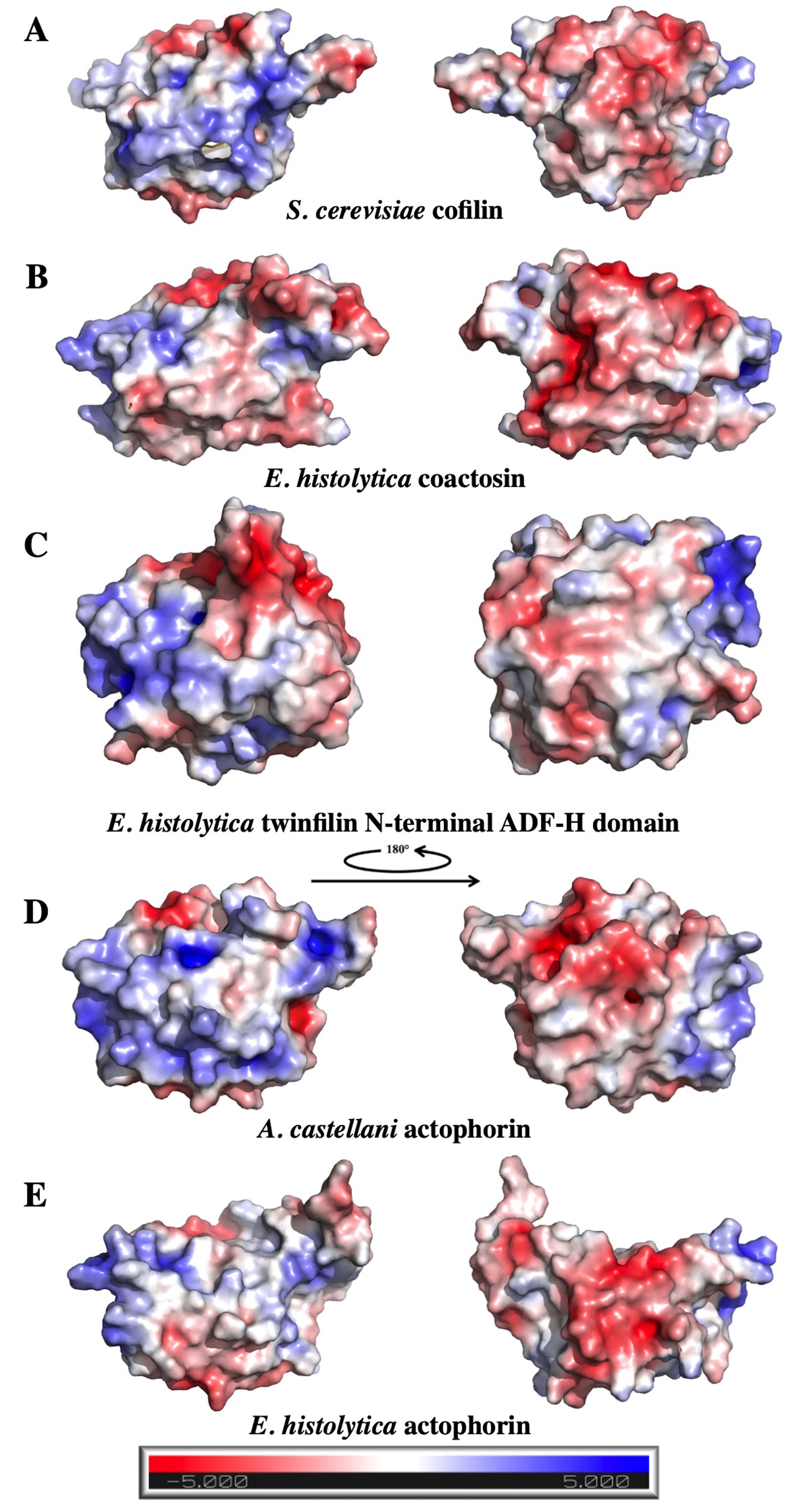

Supplement: Supplementary file 1 [file Image3.JPEG]

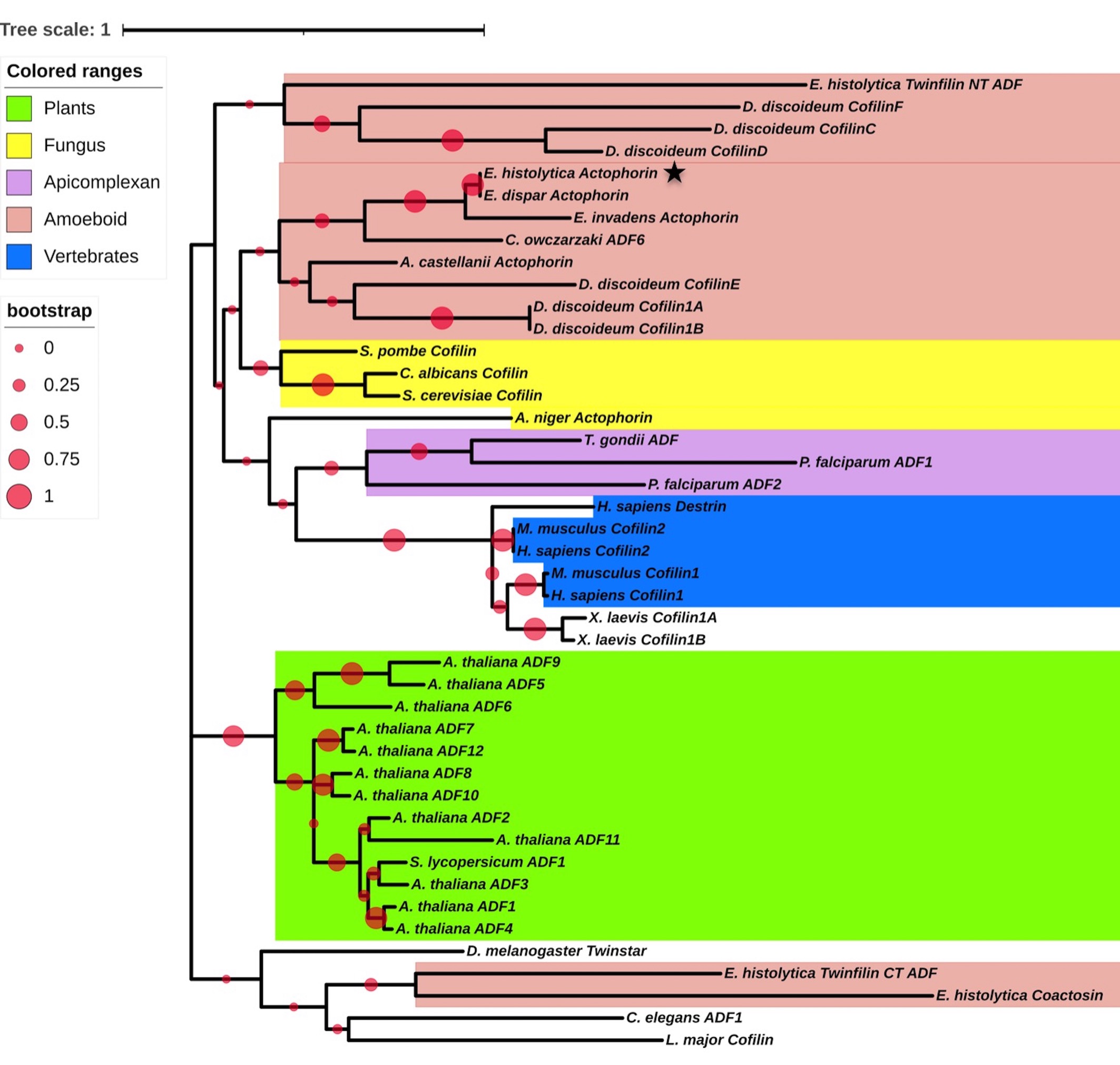

Supplement: Supplementary file 2 [file Image1.JPEG]

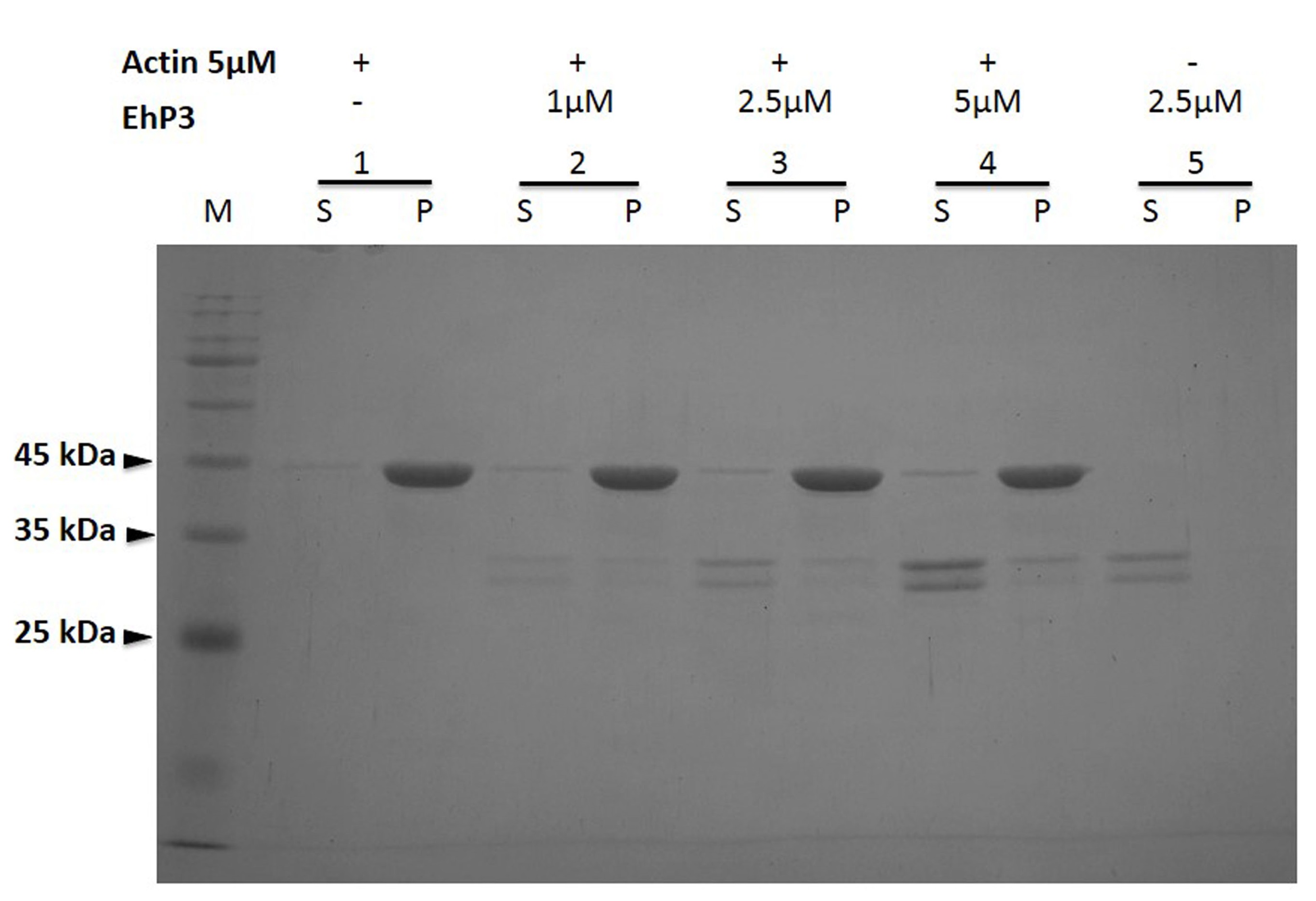

Supplement: Supplementary file 3 [file Image4.JPEG]

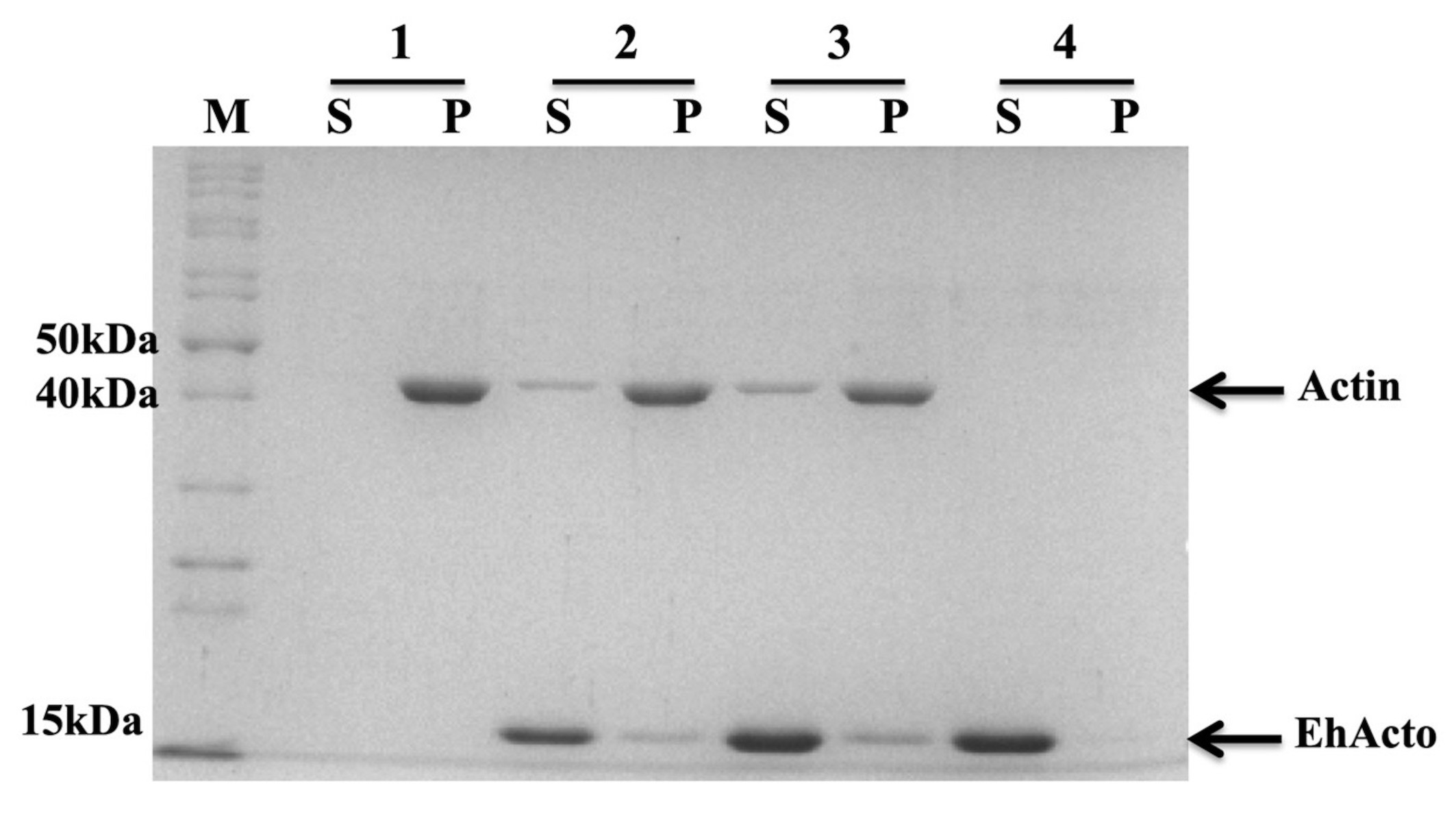

Supplement: Supplementary file 4 [file Image2.JPEG]

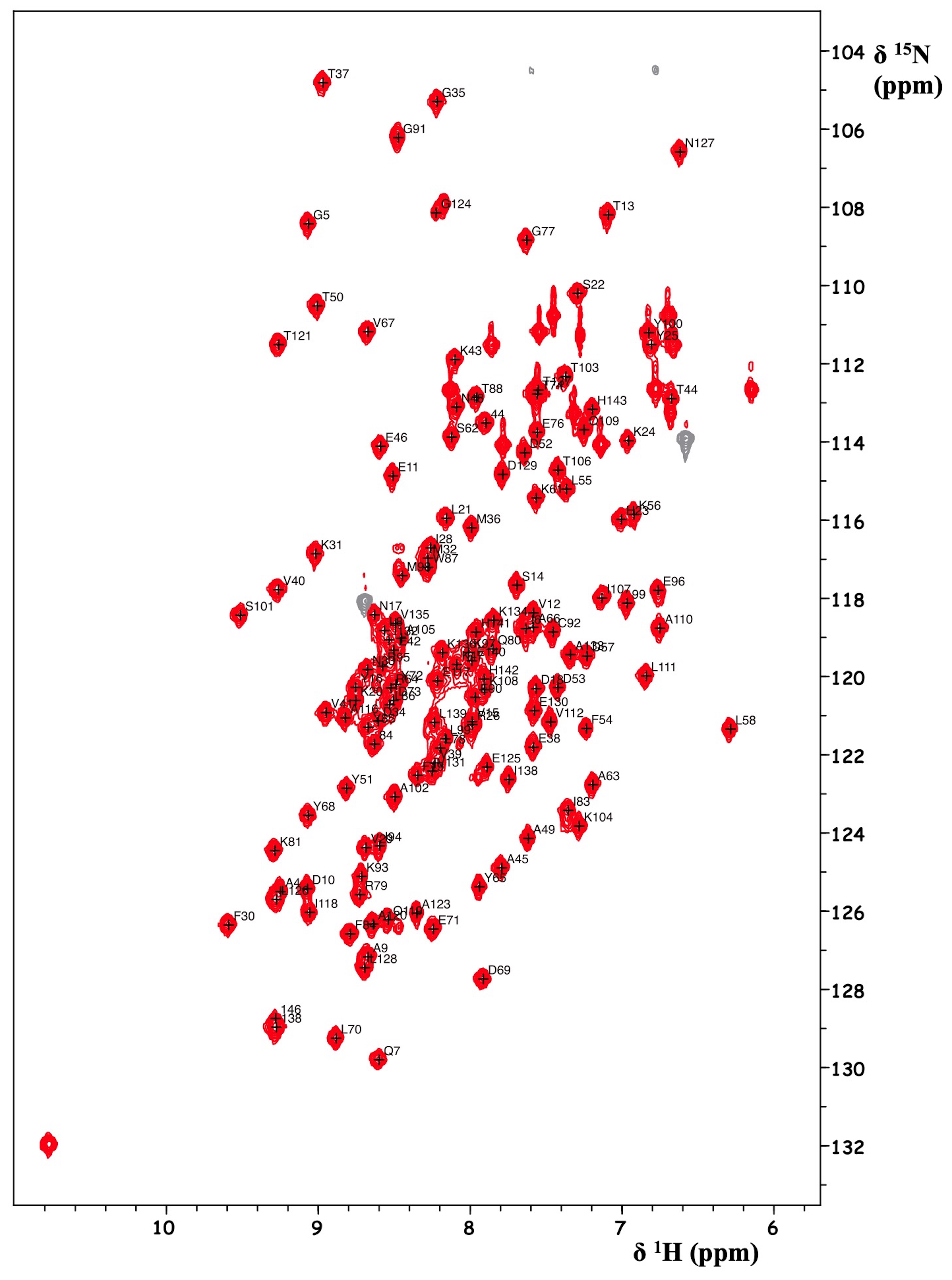

Supplement: Supplementary file 5 [file Image5.JPEG]

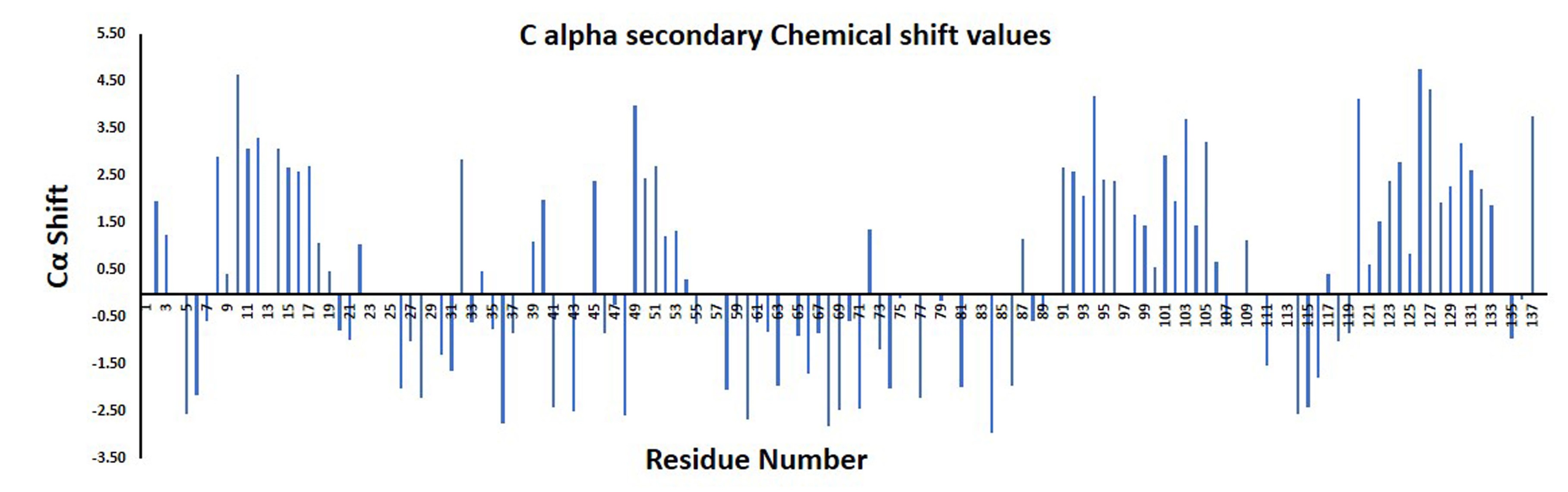

Supplement: Supplementary file 7 [file Image6.JPEG]
