# Supplementary material for: Unravelling the Biology of EhActo as the First Cofilin From Entamoeba histolytica
Source: Front Cell Dev Biol. 2022 Feb 25;10:785680. doi: 10.3389/fcell.2022.785680 (PMC8914023; doi:10.3389/fcell.2022.785680)
Supplement: Supplementary file 6 [file DataSheet1.docx]

**Supplementary method details**

M9 minimal medium preparation:

12.8 g Na_2_HPO_4_.7H_2_O / 8.5 g Na_2_HPO_4_.2H_2_O 
3.0 g KH_2_PO_4_ 
0.5 g NaCl

The above chemicals were dissolved in 900 ml water / D_2_O and the solution was autoclaved.

Post autoclaving, the following components were added to the media:
6 g D-glucose / 2.5 g ^13^C_6_-D-glucose / 2.5 g ^13^C_6_, 2H1-D-glucose 
1 g NH_4_Cl / 1 g ^15^NH_4_Cl 
1 ml Solution Q (details given below)
2 ml 1M MgSO_4_ 
The final volume was made up to 1 L with distilled H_2_O / D_2_O and the completed media was filter-sterilised.

1000X Solution Q (micronutrient mix)
8 ml HCl (fuming) 
5 g FeCl_2_.4H_2_O 
184 mg CaCl_2_.2H_2_O 
64 mg H_3_BO_3_ 
18 mg CoCl_2_.6H_2_O 
4 mg CuCl_2_.2H_2_O 
340 mg ZnCl_2_ 
605 mg Na_2_MoO_4_.2H_2_O 
40 mg MnCl_2_.4H_2_O
The volume was made up to 1 L with distilled H_2_O / D_2_O and was filter-sterilised.

**Supplementary table**

**Table S1**: List of all the sequences used for the sequence comparison and phylogenetic analysis.

| **Name of the organism** | **Sequence ID (Uniprot)** |
| --- | --- |
| *Entamoeba histolytica* Actophorin | C4LVG4 |
| *Acanthamoeba castellanii* Actophorin | L8HMC3 |
| *Entamoeba dispar* Actophorin | B0EJB0 |
| *Entamoeba invadens* actophorin | L7FML6 |
| *Capsaspora owczarzaki* ADF6 | A0A0D2VLA7 |
| *Candida albicans* cofilin | A0A1D8PMW6 |
| *Saccharomyces cerevisiae* cofilin | Q03048 |
| *Aspergillus niger* actophorin | A0A100IQQ5 |
| *Schizosaccharomyces pombe* cofilin | P78929 |
| *Solanum lycopersicum* ADF1 | A0A3Q7IMR6 |
| *Toxoplasma gondii* ADF | O15902 |
| *Caenorhabditis elegans* ADF1 | Q07750 |
| *Drosophila melanogaster* Twinstar | P45594 |
| *Xenopus laevis* Cofilin1A | P45695 |
| *Xenopus laevis* Cofilin1B | P45593 |
| *Mus musculus* Cofilin1 | P18760 |
| *Homo* *sapiens* Cofilin1 | P23528 |
| *Mus musculus* Cofilin2 | P45591 |
| *Homo* *sapiens* Cofilin2 | Q9Y281 |
| *Dictyostelium discoideum* Cofilin1A | P0DJ26 |
| *Dictyostelium discoideum* Cofilin1B | P0DJ27 |
| *Dictyostelium discoideum* CofilinC | Q966T6 |
| *Dictyostelium discoideum* CofilinD | Q556H5 |
| *Dictyostelium discoideum* CofilinE | Q54R65 |
| *Dictyostelium discoideum* CofilinF | Q86IX3 |
| *Arabidopsis thaliana* ADF1 | Q39250 |
| *Arabidopsis thaliana* ADF4 | Q9ZSK3 |
| *Arabidopsis thaliana* ADF3 | Q9ZSK4 |
| *Arabidopsis thaliana* ADF2 | Q39251 |
| *Arabidopsis thaliana* ADF9 | O49606 |
| *Arabidopsis thaliana* ADF7 | Q67ZM4 |
| *Arabidopsis thaliana* ADF12 | Q8LFH6 |
| *Arabidopsis thaliana* ADF6 | Q9ZSK2 |
| *Arabidopsis thaliana* ADF5 | Q9ZNT3 |
| *Arabidopsis thaliana* ADF8 | Q570Y6 |
| *Arabidopsis thaliana* ADF10 | Q9LQ81 |
| *Arabidopsis thaliana* ADF11 | Q9LZT3 |
| *Leishmania major* Cofilin | E9ADQ2 |
| *Plasmodium falciparum* ADF1 | Q8I467 |
| *Homo sapiens* Destrin | P60981 |
| *Plasmodium falciparum* ADF2 | Q8ID92 |

**Supplementary figures**

**Figure S1. Phylogenetic tree for several ADF/Cofilins.** Representative ADF/Cofilins from different organisms were first aligned using ClustalW and the alignments thus generated were used to trace the phylogenetic evolution of EhActo with the MEGA 7.0 software. Proteins from plants separated as one node, while those from fungal, vertebrate, apicomplexan and amoeboid origins formed one node. EhActo branched out with other amoebic ADF/Cofilins; marked with a star on the tree. The highest log-likelihood value for the tree is -5735.66.

**Figure S2. Co-sedimentation assay to analyse the interaction between EhActo and actin filaments.** Pre-polymerised actin filaments were incubated with different concentrations of EhActo for a half-hour. The samples (numbered 1-4) were then ultracentrifuged to separate the supernatant (S) and the pellet (P) fractions. Sample 1, actin control 5μM (where the entire F-actin was present in the P fraction), sample 2, 5μM actin with 10μM EhActo, sample 3, 5μM actin with 20μM EhActo, sample 4, EhActo control 20μM (where the protein was present in the S fraction only). EhActo severs/depolymerises F-actin, and thus we can see the presence of a protein band corresponding to actin in the S fraction in samples 2 and 3.

**Figure S3. Surface charge distribution profiles.** Surface charge distribution was analysed with the APBS plugin in the PyMOL suite. The charge distribution ranged, -5.0 (Red), 0.0 (White), and +5.0 (Blue). All the structures have been displayed as a set of opposite faces. (A) Yeast Cofilin has one face positively charged while the other one was negatively charged. (B) EhCoactosin has predominant negative charge around the C-terminal. (C) NT EhTWF has the most hydrophobic charge distribution range all over. (D) *Acanthamoeba* Actophorin is similar to yeast homologue. (E) The F-loop present EhActo lacks positive charges compared to others.

**Figure S4. EhP3 does not bind directly with actin.** We analysed the binding of EhP3 with actin through actin cosedimentation assay where for a constant 5μM actin, variable concentration of EhP3 was added as described in the figure.

**Figure S5**: 2D [^15^N, ^1^H] HSQC of EhActo. Assigned residues are marked with their residue number and residue name.

**Figure S6**: Cα atoms in α-helices, for example, tend to have positive secondary chemical shifts and Cα atoms in β-strands have negative secondary chemical shifts represented in the graph.
